# Prognostic value of anti-CRP antibodies in lupus nephritis in long-term follow-up

**DOI:** 10.1186/s13075-015-0879-8

**Published:** 2015-12-24

**Authors:** Satu Sinikka Pesickova, Romana Rysava, Martin Lenicek, Libor Vitek, Eliska Potlukova, Zdenka Hruskova, Eva Jancova, Eva Honsova, Jakub Zavada, Marten Trendelenburg, Vladimir Tesar

**Affiliations:** Department of Nephrology, General University Hospital and First Faculty of Medicine, Charles University, Prague, U Nemocnice 2, 12808 Prague 2 Czech Republic; Dialcorp, Hemodialysis unit, Prague, Ohradni 1368, 14000 Prague 4 Czech Republic; Institute of Medical Biochemistry and Laboratory Diagnostics, First Faculty of Medicine, Charles University, Prague, Katerinska 32, 12808 Prague 2 Czech Republic; Fourth Department of Medicine, General University Hospital and First Faculty of Medicine, Charles University, Prague, U Nemocnice 2, 12808 Prague 2 Czech Republic; Third Department of Medicine, General University Hospital and First Faculty of Medicine, Charles University, Prague, U Nemocnice 2, 12808 Prague 2 Czech Republic; Division of Internal Medicine, University Hospital Basel, Basel, Spitalstrasse 21, 4031 Basel Switzerland; Department of Pathology, Institute for Clinical and Experimental Medicine, Prague, Videnska 1958/9, 140 21 Prague 4 Czech Republic; Institute of Rheumatology, First Faculty of Medicine, Charles University, Prague, Na Slupi 4, 128 50 Prague 2 Czech Republic; Laboratory of Clinical Immunology, Department of Biomedicine, University Hospital Basel, Basel, Spitalstrasse 21, 4031 Switzerland

## Abstract

**Background:**

Autoantibodies against monomeric C-reactive protein (anti-CRP-Ab) observed in patients with systemic lupus erythematosus (SLE) and lupus nephritis (LN) were suggested to be associated with active LN and a poor response to therapy during short-term follow-up.

The aim of this study was to confirm this finding and to investigate the prognostic value of anti-CRP-Ab in patients with LN during long-term follow-up.

**Methods:**

Sera of 57 SLE patients (47 women, 10 men) with biopsy proven LN and 122 healthy individuals were analyzed for the presence of anti-CRP-Ab by in-house ELISA.

Anti-CRP-Ab levels were studied in relation to routine laboratory tests, urine analysis, levels of C3, C4, other immunological markers and the overall disease activity as assessed by Systemic Lupus Erythematosus Disease Activity Index (SLEDAI).

The prognostic value of anti-CRP-Ab was tested in a subgroup of 29 newly diagnosed LN patients (median follow-up 5.9 years). Response to therapy at various time points was assessed with respect to baseline anti-CRP-Ab levels. At least partial response in the first/second year of treatment was considered as a “favorable outcome”, while non-response, renal flare or end stage renal disease were considered as “unfavorable outcome”.

**Results:**

Anti-CRP-Ab were only detected in patients with active renal disease and their levels correlated with SLEDAI (r_s_ = 0.165, p = 0.002). The time to response was shorter in patients being anti-CRP-Ab negative at baseline compared to anti-CRP-Ab positive patients, p = 0.037. In the second year of therapy, baseline anti-CRP-Ab positivity was a significant predictor of “unfavorable outcome” (OR [95 % CI] = 15.6 [1.2-771]; p = 0.021). The predictive value of “baseline anti-CRP positivity” further increased when combined with “non-response to therapy in the first year”. Baseline anti-CRP-Ab positivity was not a predictor of “unfavorable outcome” at the end of follow-up, (OR [95 % CI] = 5.5 [0.6-71.1], p = 0.169).

**Conclusions:**

Baseline serum levels of anti-CRP-Ab seem to be a strong risk factor for a composite outcome of non-response, renal flare or end stage renal disease after two years of standard treatment of LN. The response to therapy seems to be delayed in anti-CRP-Ab positive patients.

**Electronic supplementary material:**

The online version of this article (doi:10.1186/s13075-015-0879-8) contains supplementary material, which is available to authorized users.

## Background

Systemic lupus erythematosus (SLE) is a complex autoimmune disease leading to the formation of a wide range of pathogenic autoantibodies and immune complexes. SLE mainly affects young women, with a clinically significant impact on morbidity and mortality. Renal involvement is among the most severe manifestations of SLE, which in its most aggressive forms can lead to renal failure.

The pathogenesis of SLE is only partially understood. A number of potentially pathogenic autoantibodies have been described in SLE; for example, anti-double-stranded DNA antibodies (anti-dsDNA-Ab), anti-nucleosome antibodies, and anti-C1q antibodies (anti-C1q-Ab). Some of these correlate with SLE and/or lupus nephritis (LN) activity and are used in routine clinical practice for diagnostic purposes [1–7].

Pentameric C-reactive protein (CRP) under specific conditions dissociates irreversibly into monomers (mCRP) and reveals new epitopes [8–11]. The physiological function of mCRP includes opsonization, elimination of immune complexes, and clearance of apoptotic cells. This is achieved by the interaction of mCRP with C1q and complement factor H [12].

While other acute phase proteins increase in active SLE, levels of CRP usually remain low [13]. This might be caused by a suppression of interleukin (IL)-6-mediated CRP production in hepatocytes by overexpression of interferon alpha (IFNα) [14] and by CRP gene polymorphisms [15]. Another mechanism could be an accelerated conversion of CRP into mCRP [16].

Antibodies interfering with the function of mCRP (such as IgG autoantibodies against mCRP (anti-CRP-Ab)) might lead to an altered clearance of apoptotic cells and be involved in pathogenic mechanisms of LN [12]. Interestingly, anti-CRP-Ab recognize the mCRP subunits, but not the native pentameric form of CRP, and thus—as other lupus autoantibodies—can be considered neo-epitope specific [8].

A high prevalence of anti-CRP-Ab in SLE patients was described by Bell et al. [8]. Later, anti-CRP-Ab were shown to be associated with active LN [17–19] and renal tubulointerstitial lesions [18]. Some authors observed colocalization of IgG with CRP and other factors such as C1q and anti-dsDNA-Ab in the glomerular basement membrane and the renal subendothelial space in LN [20, 21]. Another study showed that levels of anti-CRP-Ab correlated with the renal biopsy activity index, as documented on repeated renal biopsies, and predicted a poor response to therapy during an 8-month follow-up [17].

The aim of our study was to determine whether baseline anti-CRP-Ab positivity predicts the long-term outcome in LN patients treated with standard therapy.

## Methods

### Study subjects

A total of 57 patients (47 women, 10 men; median age 32.1 years) with definite SLE classified according to the American College of Rheumatology criteria [22] and biopsy-proven LN (29 new diagnoses of LN in 2005–2010, 28 previous diagnoses) were recruited into the study at the Department of Nephrology, General University Hospital, Prague, Czech Republic between 2005 and 2010. Basic patient characteristics are presented in Table 1. Renal biopsies were scored according to the classification of the International Society of Nephrology and the Renal Pathology Society in 2003 [23]. One hundred and nineteen serum samples from these patients at different time points of the disease were obtained. For comparisons of anti-CRP-Ab serum levels with those of the control group, and to correlate these levels with disease activity and other parameters, only one serum sample per patient was randomly selected.

**Table 1:** Basic characteristics of the patients

Age (years)		32 (21.7–62.4)
Gender, male/female		10/47
Caucasian/Asian		55 (96.5 %)/ 2 (3.5 %)
Renal disease duration (days)		75 (1–2413)
Renal biopsy	Class II	2 (3.5 %)
	Class III	13 (22.8 %)
	Class IV	28 (49.1 %)
	Class III/V or IV/V	11 (19.3 %)
	Undetermined class	3 (5.3 %)

Data presented as median (5th and 95th percentile) or number of patients (%)

To assess the role of baseline anti-CRP-Ab levels in the prediction of therapeutic outcome during long-term follow-up, only patients with newly diagnosed active LN (as proven by renal biopsy) were included (*n* = 29). Baseline serum samples were taken at the time of biopsy.

One hundred and twenty-two serum samples from age-matched healthy volunteers served as controls.

### Ethics

The study protocol was approved by the Ethics Committee of the General University Hospital, Prague (2087/10 A, D; basic study 706/04 S) and made to conform to the ethical guidelines of the latest Declaration of Helsinki. Informed consent was obtained from all subjects.

### Definitions

Global disease activity was assessed by the Systemic Lupus Erythematosus Disease Activity Index (SLEDAI) [24]. In this study, active LN was defined as active urinary sediment, and/or proteinuria ≥0.5 g/day, and/or worsened glomerular filtration rate (GFR) >25 % above baseline/normal range caused by active LN, and/or C3 hypocomplementemia while any other causes were excluded. At least two of the aforementioned criteria had to be met.

Response to therapy at follow-up was assessed according to European consensus criteria [25].

Complete response was defined as inactive urinary sediment, decrease of proteinuria to ≤0.2 g/day, and normal/stable renal function (<10 % of normal GFR). Partial response was defined as inactive urinary sediment, proteinuria ≤0.5 g/day, and normal/stable renal function (<10 % from baseline levels if abnormal).

At least partial response in the first/second year of treatment was considered a “favorable outcome”, while nonresponse, renal flare, or end-stage renal disease (ESRD) was considered an “unfavorable outcome”.

Renal flare was defined as an increase of disease activity requiring more intensive therapy (addition/change of immunosuppressive therapy, or administration of high-dose pulses of corticosteroids).

### Biochemical analyses

At the time of blood sampling, routine laboratory tests in all patients were performed in the laboratories of the Institute of Medical Biochemistry and Laboratory Diagnostics of the General University Hospital, Prague, including measurements of 24-hour proteinuria, GFR, urinary sediment analyses, complement C3 and C4, and anti-dsDNA-Ab. Further analyses were carried out subsequently from sera stored at −80 °C. Serum amyloid A (SAA) was determined by a commercial enzyme-linked immunosorbent assay (ELISA) kit (Invitrogen, Carlsbad, CA, USA), high-sensitivity CRP (hsCRP) was measured by particle-enhanced immunonephelometry (Behring Nephelometer II; Siemens, Munich, Germany), and anti-C1q-Ab were measured by ELISA (Bühlmann Laboratories, Switzerland. Schönenbuch).

### Anti-CRP-Ab assay

Levels of anti-CRP-Ab were measured by in-house ELISA according to Sjöwall et al. [26] with slight modifications. One hundred microliters of human native CRP (Sigma, St. Louis, MO, USA) diluted with carbonate/bicarbonate buffer (50 mM, pH 9.6) to a concentration of 1 mg/l was added to each well of a Maxisorp microtiter plate (Nunc, Denmark, Roskilde) and incubated overnight at room temperature. After washing four times with phosphate-buffered saline (PBS, pH 7.4) with 0.05 % Tween 20 (Sigma), coated plates were incubated (1 hour, room temperature) with 100 μl serum samples diluted 1:20 with PBS–Tween. After an additional four-times wash with PBS–Tween, 100 μl alkaline phosphatase-conjugated rabbit IgG antibodies raised against human γ-chains (DAKO, Denmark, Glostrup) was added (diluted 1:500 in PBS–Tween). After 1 hour of incubation, plates were washed four times with PBS–Tween and 100 μl *p*-nitrophenyl phosphate (10 g/l; Sigma) diluted in the reaction buffer (carbonate/bicarbonate buffer, 50 mM, pH 9.6; 1 mM MgCl_2_) was added. After 1 hour of incubation (room temperature, dark), the reaction was stopped by addition of 25 μl of 3 M NaOH. Absorbances were read at 405 nm. All samples were analyzed in quadruplicate. A calibration curve was constructed by serial dilution of the most positive sample with the negative one. Results were expressed as arbitrary units (AU), which are defined as a percentage of the highest patient sample. The cutoff value for a positive test (45.5 AU) was set as the 95th percentile in 122 healthy individuals. The limit of detection was 15 AU.

### Statistical analyses

Owing to the skewed data distribution, nonparametric tests were used. Results are presented as the median plus 5th and 95th percentiles, numbers (%), or odds ratio (OR) and 95 % confidential interval (95 % CI). The predictive value of two risk factors (baseline anti-CRP-Ab positivity and nonresponse in the first year) was assessed using logistic regression. To prevent model overadjustment (the small number of subjects enabled us to reliably analyze only one predictor), both predictors were combined to form a single risk factor. Because they have similar ORs, both predictors were considered equipotent and were used unweighted.

Furthermore, the Mann–Whitney rank sum test, Kruskal–Wallis analysis of variance and Spearman’s correlation coefficient (*r*_s_) were calculated and Kaplan–Meier survival curves were constructed using STATISTICA software (version 9; StatSoft Inc., Tulsa, OK, USA). Differences between survival curves were tested by the Mantel–Cox test. Fisher’s exact test, the chi-square test, and logistic regression were calculated using EpiInfo (version 3.5.3; Centers for Disease Control and Prevention, Atlanta, GA, USA). For calculations, an arbitrary anti-CRP-Ab concentration of 7.5 AU was assigned to each sample that was below the limit of detection (15 AU).

## Results

### Prevalence of anti-CRP-Ab and their relationship to SLE activity

Levels of anti-CRP-Ab were significantly higher in LN patients compared with healthy controls (21.1 AU (<15.0–98.6) vs. <15.0 AU (<15.0–45.5), respectively; *p* = 0.012). Over one-quarter (26.3 %) of SLE patients was considered to be anti-CRP-Ab-positive (i.e., the concentration exceeded the threshold value of 45.5 AU). Anti-CRP-Ab positivity was exclusively observed in patients with active LN 15/46 (33 %), while it did not occur in patients with inactive renal disease (0/11, *p* = 0.051). In addition, levels of anti-CRP-Ab were significantly higher in patients with active LN than in patients with inactive LN (26.8 AU (<15.0–89.4) vs. <15.0 AU (<15.0–30.6); *p* = 0.009) and correlated with the overall activity of SLE as assessed by the SLEDAI (*r*_s_ = 0.41, *p* = 0.002).

### Anti-CRP-Ab association with established immunological markers of LN

A significant negative correlation was found between levels of anti-CRP-Ab and complement C3 (*r*_s_ = − 0.509, *p* < 0.0001), but not complement C4. Additionally, anti-dsDNA-Ab-positive patients had significantly higher levels of anti-CRP-Ab as compared with anti-dsDNA-Ab-negative patients (31.6 AU (<15.0–91.6) vs. <15.0 AU (<15.0–57.8); *p* = 0.007). However, the levels of anti-CRP-Ab did not correlate with anti-C1q-Ab (*r*_s_ = 0.127, *p* <0.353).

There was also no association between anti-CRP-Ab levels and the acute phase proteins SAA and hsCRP.

### Clinical follow-up

Twenty-nine patients with newly diagnosed active LN were followed for a median of 5.9 (3.9–7.4) years. A baseline comparison of anti-CRP-Ab-positive and anti-CRP-Ab-negative patients is summarized in Additional file 1.

During follow-up, one patient died, two were lost from follow-up, and two patients were excluded because of noncompliance to therapy. Additionally, one patient’s data could not be analyzed because he did not fulfill criteria either for “favorable” or for “unfavorable” at year 2. Data from 26 patients were available at year 1, while we obtained data from 25 patients at year 2. Data from 23 patients were available for analysis at the end of follow-up (March 2014). An overview of induction and maintenance therapy is presented in Additional file 1.

**Fig. 1:**
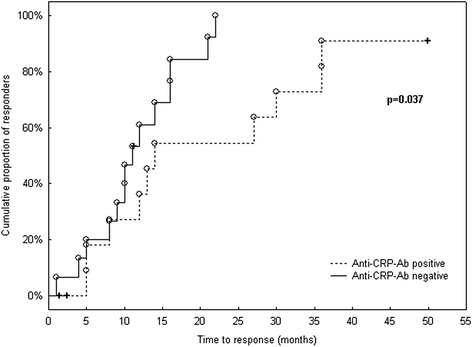
Time to response in anti-CRP-Ab-positive/negative LN patients. Kaplan–Meier survival curves demonstrate faster achievement of response in baseline anti-CRP-Ab-negative LN patients. *Crosses* indicate censored patients (i.e*.* those not responding within the follow-up period or lost from follow-up; *n* = 4), while finished patients (i.e. responders; *n* = 24) are shown as *open circles. anti-CRP-Ab* anti-C-reactive protein antibodies

**Fig. 2:**
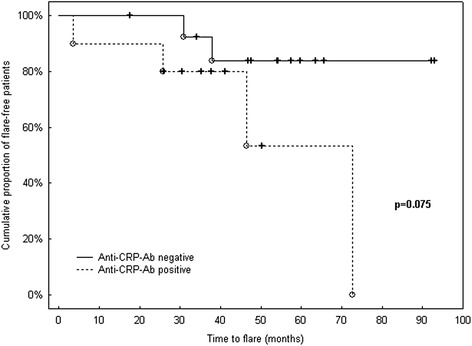
Time to flare in anti-CRP-Ab-positive/negative LN patients. Kaplan–Meier survival curves demonstrate time to flare in baseline anti-CRP-Ab-positive/negative LN patients. *Crosses* indicate censored patients (i.e. those who had not experienced flare within the follow-up period or those lost from follow-up; *n* = 18), while finished patients (i.e. patients with flare; *n* = 6) are shown as *open circles. anti-CRP-Ab* anti-C-reactive protein antibodies

Most interestingly, the time to response was significantly shorter in baseline anti-CRP-Ab-negative patients than in those anti-CRP-Ab-positive (*p* = 0.037) (Fig. 1). In addition, after having reached partial/complete response, the time to flare tended to be shorter in anti-CRP-Ab-positive than in anti-CRP-Ab-negative patients, although this difference did not reach statistical significance (*p* = 0.075) (Fig. 2).

During the long-term follow-up period, 5/10 (50 %) of baseline anti-CRP-Ab-positive patients experienced at least one flare after previous response, while only 2/13 patients (15.4 %) had renal flare in the other group. This difference, however, was not significant, most likely due to the low number of patients involved (OR (95 % CI) = 5.5 (0.6–71.1), *p* = 0.169).

### Predictive value of anti-CRP-Ab

After the first year of therapy, 6/11 (54.5 %) baseline anti-CRP-Ab-positive patients and 6/15 (40 %) anti-CRP-Ab-negative patients did not respond to therapy (*p* = 0.736).

**Fig. 3:**
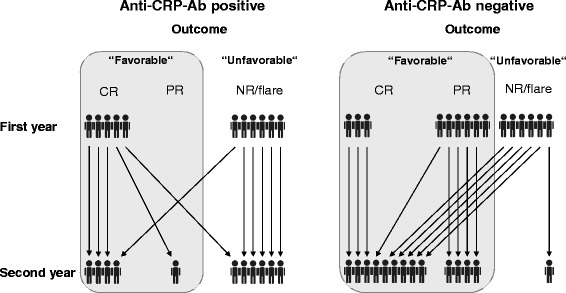
Response to therapy in anti-CRP-Ab-positive/negative LN patients. Baseline anti-CRP-Ab-positive patients are more likely to exhibit “unfavorable outcome” after 2 years of therapy, when compared with anti-CRP-Ab-negative patients*. anti-CRP-Ab* anti-C-reactive protein antibodies, *CR* complete response, *NR* nonresponse, *PR* partial response

However, at the end of the second year of follow-up, 6/11 (54.5 %) baseline anti-CRP-Ab-positive patients had “unfavorable outcome” (nonresponse to therapy or renal flare), while only one patient remained without response in the baseline anti-CRP-Ab-negative group (9.1 %) (see Fig. 3). Baseline anti-CRP-Ab positivity was thus a significant predictor of “unfavorable outcome” (OR (95 % CI) = 15.6 (1.2–771); *p* = 0.021).

As described previously, early response to treatment is associated with better outcome [27, 28]. This was also the case in our cohort: patients who did not respond to therapy in the first year were more likely to exhibit “unfavorable outcome” in the second year (OR (95 % CI) = 12 (1–596.6); *p* = 0.03). Because these two predictors (baseline anti-CRP-Ab positivity and nonresponse in the first year) seemed to be independent (not correlating), we tested whether they might have an additive effect. Our data show that each of the risk factors increased the risk of “unfavorable outcome” more than 20 times (OR (95 % CI) = 26.3 (2.2–308.7); *p* = 0.009) and the OR in patients having both risk factors would be doubled.

## Discussion

The occurrence of anti-CRP-Ab has been described previously, most often in patients diagnosed with SLE [8, 18, 19, 29–32]. In our study, we focused on patients with LN proven by renal biopsy. We found that prevalence of anti-CRP-Ab in newly diagnosed active LN was 44.8 % and that anti-CRP-Ab positivity was significantly associated with “unfavorable outcome” at 2 years. The therapeutic response in anti-CRP-Ab-positive patients was delayed.

Published prevalence of anti-CRP-Ab in patients with SLE varied between 26 % and 78 % [8, 17–19, 26, 29, 31–36]. Generally, anti-CRP-Ab appear mostly in patients with active disease, especially in those with renal involvement [17–19]. In our study, approximately one-quarter of LN patients were anti-CRP-Ab-positive. Because anti-CRP-Ab positivity was observed exclusively in patients with active renal disease, it appears that anti-CRP-Ab could serve as a useful marker of active LN. This is in accordance with conclusions of Sjöwall et al. [17], who observed a decrease of anti-CRP-Ab in patients with active renal disease in transition to remission. Additionally, we can confirm that anti-CRP-Ab levels correlate with the activity of SLE [17, 18, 29].

Besides monitoring SLE disease activity, anti-CRP-Ab might exhibit an even more important role; that is, a predictive potential as suggested by Sjöwall et al*.* [17], who reported more than a doubled risk for adverse therapeutic response in patients with baseline anti-CRP-Ab positivity. We could not confirm anti-CRP-Ab as a significant predictor of “unfavorable therapeutic outcome” after 1 year of therapy (OR = 1.8; *p* = 0.368). However, as described previously, the response to therapy in LN patients is relatively slow and many patients only achieve remission after the switch to “maintenance” therapy [37]. We now show that this may be especially true in anti-CRP-Ab-positive patients who showed longer time to response than anti-CRP-Ab-negative patients, which might have potential therapeutic consequences.

Because of the possible delay of a therapeutic response in SLE, we assessed the therapeutic response also after 2 years of standard therapy. Additionally, we were monitoring the frequency of flares throughout the follow-up (median 5.9 years).

After 2 years of therapy, during the time of maintenance therapy, baseline anti-CRP-Ab positivity seemed to be a strong predictor of therapeutic response. Positive patients faced a more than 10 times higher risk of nonresponse when compared with those being anti-CRP-Ab-negative. Not surprisingly, the therapeutic response after 2 years of therapy depended on the response in the first year. Again, patients not responding in the first year are more likely (more than 10 times) not to respond during prolonged therapy. The predictors already mentioned seem to be independent, each having an additive effect. Patients carrying both risk factors (anti-CRP-Ab-positive at baseline, not responding at year 1) are at approximately 50 times higher risk of treatment failure in the second year. This strong prediction thus suggests that in such patients intensification of existing therapies or an alternative therapeutic approach (e.g. biological therapy) should be considered.

In the extended follow-up period, anti-CRP-Ab were not confirmed as a significant predictor of renal flares (OR = 5; *p* = 0.092). However, the quite high OR and borderline significance do not allow us to reject anti-CRP-Ab positivity as a predictor of flares, and an independent confirmative study recruiting a larger number of patients with prospective follow-up seems to be needed to draw a final conclusion.

To the best of our knowledge, this is the first study assessing the predictive role of anti-CRP-Ab in the long-term follow-up. The relatively small number of patients (accompanied by quite low statistical power of used tests) is probably the major limitation of this study. As a result, calculated ORs are imprecise (95 % CIs are very wide) and several interesting findings could not be considered as being significant. Because of the low frequency of the disease, a multicenter study seems to be essential. Another limiting factor was the unavailability of repeated renal biopsy samples—the response to therapy (and activity of LN) was assessed only by means of laboratory and clinical investigation. However, repeated renal biopsies are invasive and not routinely performed to date.

## Conclusion

Our data indicate that anti-CRP-Ab positivity might be a strong predictor of unfavorable long-term therapeutic response, which, moreover, seems to be delayed. Baseline anti-CRP-Ab-positive patients, especially those not responding to standard therapy within 1 year, seemed not to benefit from further standard treatment. Nevertheless, the potential role of the routine use of anti-CRP-Ab measurement for the monitoring and guidance of treatment in LN patients needs to be confirmed in larger prospective studies.

## Additional file

Additional file 1:
**is a table presenting baseline characteristics and laboratory data of patients with newly diagnosed LN.** Data presented as median (5th and 95th percentiles) or number (%), differences compared using the Mann–Whitney test unless otherwise stated. *Fisher Exact test. **Expressed as number (%) of patients with erytrocyturia above normal range. ***χ^2^, GFR expressed as clearance of creatinine. *Anti-CRP-Ab* anti-C-reactive protein antibodies, *anti-C1q-Ab* anti-C1q antibodies, *GFR* glomerular filtration rate, *hsCRP* high-sensitivity C-reactive protein, *NS* not significant, *SLEDAI* Systemic Lupus Erythematosus Disease Activity Index. (XLS 17 kb)

## Abbreviations

anti-CRP-Ab: Anti-C-reactive protein antibodies
anti-C1q-Ab: Anti-C1q antibodies
anti-dsDNA-Ab: Anti-double-stranded DNA antibodies
AU: Arbitrary unit
CI: Confidence interval
CRP: C-reactive protein
ELISA: Enzyme-linked immunosorbent assay
ESRD: End-stage renal disease
GFR: Glomerular filtration rate
hsCRP: High-sensitivity C-reactive protein
IFNα: Interferon alpha
IL: Interleukin
LN: Lupus nephritis
mCRP: Monomeric C-reactive protein
OR: Odds ratio
r_S_: Spearman’s correlation coefficient
PBS: Phosphate-buffered saline
SAA: Serum amyloid A
SLE: Systemic lupus erythematosus
SLEDAI: Systemic Lupus Erythematosus Disease Activity Index
